# Ten quick tips to SNIFF out sustainable and secure scientific software

**DOI:** 10.1371/journal.pcbi.1014510

**Published:** 2026-07-15

**Authors:** V. P. Nagraj, Karsten H. Siller, Thomas Stewart, Neal Magee, Stephen D. Turner

**Affiliations:** 1 University of Virginia School of Data Science, Charlottesville, Virginia, United States of America; 2 University of Virginia Comprehensive Cancer Center, Charlottesville, Virginia, United States of America; Montreal, CANADA

## Abstract

Modern computational biology depends heavily on open-source software tools, analysis pipelines, and containerized workflows developed and shared by the research community. While there is extensive guidance (including Quick Tips and Simple Rules articles) on how to build robust and sustainable scientific software, far less has been written for researchers in the role of software users evaluating whether an existing tool is reliable, secure, and sustainable enough for their work. Here we present ten quick tips to help researchers critically assess the tools they adopt. Our tips are organized around a framework that centers on key evaluation features: source, network, interaction, fit, and fragility (SNIFF). These dimensions prompt researchers to consider who maintains a tool and why, whether it is embedded in a broader ecosystem, how actively its developers and users engage, whether it matches the intended use case and licensing requirements, and how robust its dependencies and security practices are. By applying these tips, researchers can make more informed decisions, reduce the risk of relying on abandoned or insecure software, and contribute to a more sustainable scientific software ecosystem.

## Introduction

Modern computational biology relies on open-source software tools, analysis pipelines, and containerized workflows to process large datasets and perform complex analyses [1]. Platforms like GitHub and container technologies such as Docker have made it routine to share not only code but entire computational environments, allowing researchers to distribute fully reproducible workflows [2]. This open and community-focused approach has accelerated discovery, but it also means that researchers are increasingly using tools they did not personally develop. The question, “Can I trust this tool?”—in terms of its results, security, and longevity—has become important as science leans on community-created software.

Over the past decade, many PLOS Computational Biology articles have offered guidance to scientific software developers on how to build sustainable, usable, and robust tools. The term “sustainable” in a software context denotes code that is reliable, reproducible, and reusable, ensuring a project’s long-term health, as distinct from environmental sustainability [3]. The popular Ten Simple Rules and Quick Tips series cover best practices in open-source software design, documentation, usability, version control, testing, and reproducible workflows [1,2,4–7]. For example, there are Ten Simple Rules for reproducible computational research [8], writing clean and usable code [9], for making research software more robust and portable [10], for writing Dockerfiles to enable reproducible analyses [2], and for making computing practices more environmentally sustainable [3]. Community initiatives have also emphasized training scientists in better software engineering practices [1,11,12] and adopting standards like FAIR (Findable, Accessible, Interoperable, Reusable) for workflows [13]. In short, there is a rich body of literature and active community efforts to improve software, mostly aimed at developers of tools.

However, there is far less guidance for researchers in the role of software *users*—those trying to infer whether an existing tool is trustworthy, secure, and sustainable enough to incorporate into their own work. Many scientists have discovered the hard way that using someone else’s code can be fraught with issues: the software may be poorly documented, behave unpredictably (if it runs at all outside the original developer’s setup), rely on obsolete dependencies, or be an outdated version that was never updated after publication [10]. In bioinformatics and other fields, it’s common to find multiple tools for the same task, and a substantial fraction of these fail to be maintained or supported after their initial release [14–16]. Some guidance exists for specific ecosystems, such as strategies for discovering and selecting R packages [17], but the literature largely lacks a language-agnostic framework for evaluating a tool’s long-term reliability, security posture, or community support before investing time in it. As a result, one might choose a pipeline only to later realize that its container image hasn’t been updated for years, or that it harbors a security vulnerability, or that no one can answer usage questions.

This article aims to fill that gap by providing guidance on evaluating the sustainability and security of scientific software at the point of adoption. We draw inspiration from community efforts like Bioconductor [18] and the Galaxy Project [19] that have built thriving ecosystems around sustainable software practices. Bioconductor maintains a curated repository of over 2,000 R packages for genomic data analysis with standardized documentation, rigorous testing, and transparent peer review. Similarly, the Galaxy Project fosters community activities to build its framework and establishes clear governance, dedicated working groups, and documentation on code of conduct and ways to contribute. Galaxy maintainers have formalized mechanisms for peer review of workflows and the Galaxy ToolShed through the Intergalactic Workflow Commission and Intergalactic Utilities Commission, respectively. By contrast, many tools outside such communities require extra scrutiny, especially in an era when AI-generated code is on the rise, raising new questions about software provenance and hidden security flaws. While these tips are grounded in examples from computational biology and bioinformatics, the principles apply broadly to any domain where researchers rely on externally developed software.

**Fig 1:**
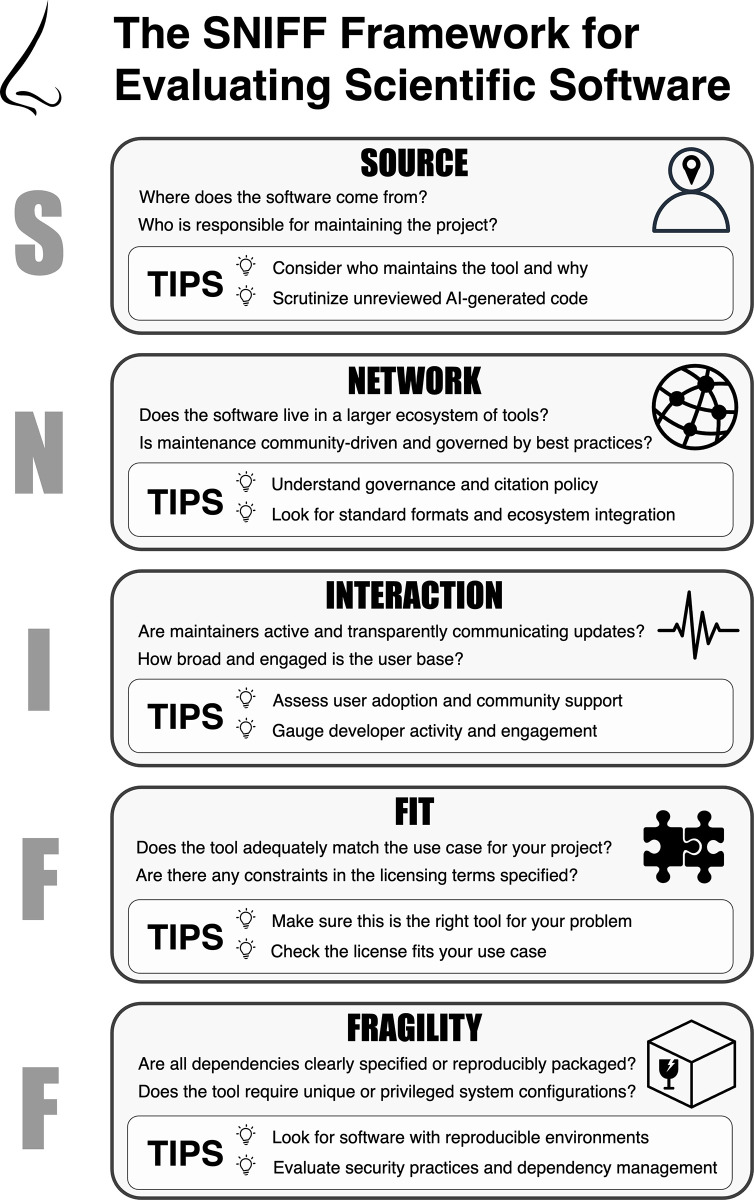
The SNIFF framework organizes ten quick tips for evaluating scientific software sustainability and security into five complementary dimensions: Source, Network, Interaction, Fit, and Fragility.

In the tips that follow, we present strategies to proactively assess the reliability of a tool. These strategies center on key features that we have summarized as *source*, *network*, *interaction*, *fit*, and *fragility* (SNIFF; see Fig 1):

- **Source:** Where does the software come from? Who is responsible for maintaining the project?
- **Network:** Does the software live in a larger ecosystem of tools? Is maintenance community-driven and governed by best practices?
- **Interaction:** Are maintainers active and transparently communicating updates? How broad and engaged is the user base?
- **Fit:** Does the tool adequately match the use-case for your project? Are there any constraints in the licensing terms specified?
- **Fragility:** Are all dependencies clearly specified or reproducibly packaged? Does the tool require unique or privileged system configurations?

The SNIFF framework is deliberately framed as a set of guiding principles rather than a checklist of heuristics. Sustainability signals may not always align cleanly. A tool might be highly cited but poorly documented; part of a large, well-governed network of tools but built on a delicate dependency stack; or pristinely packaged but kept alive by a single, overworked maintainer. For some tips, it is possible to arrive at a binary determination. But for others, the relevant signal may be fuzzy and best weighed against other indicators. Choosing a sustainable tool is therefore less about scoring candidates on each axis and more about stepping back to ask whether the collected signals, taken together, suggest whether a tool will be usable, trustworthy, and supported moving forward.

Our goal in expressing the SNIFF features as a set of Quick Tips is to empower researchers to make informed decisions about the tools they rely on. By considering factors like a project’s active support, update history, licensing, packaging techniques, community endorsements, and security practices, scientists can mitigate risks and choose tools that stand the test of time. We acknowledge that in sufficiently specialized subfields, the “right” tool may simply be the only tool. In this light, SNIFF serves less as a comparison framework and more as a guide to understanding implicit sustainability risks. As computational biology continues to depend on openly available software, looking for signs of sustainable and secure tooling is increasingly essential. These tips will help ensure that the next analysis you run is not only innovative, but that it’s also reproducible, secure, and sustainable for the long haul.

## Tip 1. Make sure this is the right tool for your problem

Computational biology tools evolve quickly, and for many tasks there are multiple viable options. Choosing a tool that genuinely fits your problem is therefore critical, and that choice depends on understanding the full scope of what you are trying to solve. When you first find a tool, make sure it’s doing what you expect. Documentation, example usage, and any associated manuscripts can help. If you’re still not sure, try contacting the maintainer directly. The timeliness, clarity, and technical depth of the response can be strong indicators as to whether the tool is a good fit. It also helps to step back and consider how your requirements may evolve as your analysis or software matures. For example, you might initially select a variant caller for germline analysis on a small cohort, only to later need somatic variant calling from tumor-normal pairs. Some callers support both modes; others are strictly designed for one, requiring you to adopt and validate an entirely new tool mid-project. The tools you select should be flexible enough to accommodate both present and future needs. This up-front “suitability check” ideally also includes a review of the tools’ computational requirements (memory, storage and I/O, CPU or GPU) and how they align with the infrastructure available to you, before they become constraints.

## Tip 2. Check that the license fits your use case

Software licenses are important, yet it’s still quite common to find bioinformatics tools that fail to clearly specify a license [20–22]. For users of open-source software, understanding and respecting developers’ intentions for how their work may be used is critical. Licensing terms should therefore be identified at the earliest stages of tool evaluation (e.g., check for a LICENSE file at the repository root or a license field in the package manifest). Fortunately, there are excellent, freely available resources that can help interpret how different licenses may affect your intended usage [23–25]. Proceeding without this awareness can lead to costly downstream consequences, such as needing to refactor code to replace a dependency whose license is incompatible with your institution’s intellectual property policies, or negotiating directly with a maintainer to alter licensing terms. Both scenarios are time-consuming and can complicate how, or whether, your work can ultimately be shared.

## Tip 3. Consider who maintains the tool and why

Longevity and sustainability of bioinformatics tools critically depend on their maintenance and support model. Open-source software may be maintained by individuals, organizations, or communities. The type of maintainer often reflects the level of support available. Broader developer involvement (e.g., a contributor list with two or more developers active in the past year, or an explicit acknowledgment of grant or institutional core support) is often preferred as it generally leads to faster bug fixes, more regular feature development, and stronger long-term maintenance. Furthermore, an open collaborative project spreads the maintenance and improvement workload across many people, which greatly enhances sustainability [26]. When possible, check the documentation for signs of funding that supported the project’s initial development or ongoing maintenance. It is also useful to look at other software produced by the same maintainer to evaluate patterns of upkeep. Projects with indications of ongoing funding support and maintainers who consistently exhibit sustainable practices are preferable as well.

## Tip 4. Assess user adoption and community support

The needs of a large and engaged user base can drive maintenance activities, and metrics can reveal how widely a tool is used in the wild. Software released in standard repositories will have usage statistics (e.g., Bioconductor downloads) or other popularity signals (e.g., GitHub stars). Citation patterns are also useful when assessing user adoption. An active user community, along with regularly used support channels such as mailing lists, Slack, or GitHub Issues, is a hallmark of sustainable software. Also, look at how responsive the developers are to bug reports or questions; timely, helpful responses are indicators of ongoing support. Open-source projects thrive on community engagement: they tend to build trust and innovation by involving users in feedback and contributions [27]. It is worth acknowledging that indicators of usage and popularity can take time to manifest, as there may be a lag between release and uptake. Likewise, overreliance on metrics can incentivize developers to “game” or selectively broadcast certain popularity signals. However, generally if you see evidence of a large and engaged community, you can be more confident in future support.

## Tip 5. Gauge developer activity and engagement

Evaluate the project’s development activity to ensure the software isn’t a “one-and-done” release. On platforms like GitHub or GitLab, look at the commit history and release timeline. A sustainable tool will have recent commits, version updates, or issue discussions indicating ongoing improvements. Useful rough heuristics include a commit within the past year, recent issues that received a maintainer response within roughly two weeks, and at least one pull request merged from a contributor outside the original author group. Many tools are unfortunately abandoned right after their paper is published, leaving users without support or fixes. An actively maintained project, on the other hand, will show a history of bug fixes and responses to user feedback. Besides signaling sustainability, regular contribution activity has been shown to be indicative of software accuracy [14]. In short, if the last update was several years ago or the issue tracker is full of unanswered problems, the tool’s longevity and reliability are questionable.

## Tip 6. Understand governance and citation policy

Healthy projects often have documented governance (e.g., a GOVERNANCE.md or CONTRIBUTING.md file, defined maintainer roles, or a code of conduct for contributors) indicating that the authors have thought about long-term project life. Look for signs that maintainers are seeking input from a wider development community. Additionally, reputable scientific software will usually tell you how to cite it (often via a DOI for a software repository, a CITATION.cff file at the repository root, or a reference to a journal article). In fact, many community registries [28–30] include metadata such as the software’s license, version, and citation info (recommendation #2 in [27]). The presence of a citation guideline shows the authors expect and welcome others to use their tool in publications. In short, a governance structure and citation policy signal that the tool is meant to be extended, shared, and credited in the scientific community.

## Tip 7. Prefer software packaged with reproducible environments

Sustainable tools make it easy to recreate the exact software environment needed to run them. This often means providing a container image (e.g., Docker or Apptainer) or environment specification file. However, container images themselves require maintenance. An image built years ago may contain outdated or vulnerable base layers (see Tip 9). Containerization can eliminate the “it works on my machine” problem by packaging all dependencies and system setup into a portable image. Likewise, look for tools that supply Conda environment files or lists of package requirements (e.g., a requirements.txt for Python, or renv lockfile for R) with specific version numbers. Such reproducible environments let you deploy the software reliably on different systems with minimal configuration effort [13]. If a tool offers a Docker/Apptainer image or a Binder environment, that’s a strong positive sign: it means the authors cared about easing installation and ensuring consistent behavior across platforms. An even stronger sign is evidence that the image is rebuilt regularly, such as a CI workflow that triggers on dependency updates or container tags published within the past year.

## Tip 8. Check for standard formats and ecosystem integration

Give preference to software that adheres to established standards in its domain and fits into common workflows. Tools that use standard data formats for input/output (for example, common file types in genomics or image analysis) will interoperate more easily with other tools [10]. Similarly, if a tool is built using a widely adopted workflow engine or framework (such as Nextflow [31], Snakemake [32], or Galaxy [19]), it’s more likely to be portable and reproducible in different environments. A great example is the nf-core initiative [33] (e.g., the nf-core/rnaseq pipeline [34]), which provides community-curated pipelines following strict guidelines. All nf-core pipelines must meet consistent standards to ensure reproducibility, interoperability, and portability. Software that is part of such frameworks or that follows community best practices is usually better vetted and easier to adopt than ad-hoc, standalone scripts.

## Tip 9. Evaluate security practices and dependency management

Security may not be a researcher’s first concern, but understanding basic best practices can help prevent serious problems. One aspect to evaluate is how the software manages its dependencies. Does it clearly list the external libraries or packages it relies on, ideally with specific versions? Understanding this dependency tree is important not only for reproducibility but also for assessing supply chain risk, as vulnerabilities in upstream packages can propagate to all downstream tools that depend on them. For example, many Python projects include a requirements.txt or Conda environment file that enumerates required packages and versions (Rule #6 in [10]). This transparency means you can identify whether those dependencies are widely used, up-to-date libraries, which tend to be safer than depending on obscure or unmaintained ones. It is also critical to assess any necessary system configurations to run the software. Tools that require disabling security features or run with elevated privileges should raise concerns, as one small bug or malicious code could do outsized damage (Rule #7 in [10]). Example red flags include install instructions that begin with sudo, requirements to disable SELinux, or containers that must run as root. Software that requires keys or credentials but manages them in plain text should also be used with caution. In practice, choosing a tool that follows basic security hygiene will reduce the likelihood of vulnerabilities affecting your research.

## Tip 10. Scrutinize unreviewed AI-generated code

The quality of the code itself matters for long-term trustworthiness. As a researcher, you might not read every line of code, but there are clues to whether the software has been carefully reviewed and tested. Look at the project’s repository (if available) for evidence of code reviews or testing. For instance, multiple contributors discussing changes, human discussion on pull requests, or the presence of unit tests and continuous integration badges. If all code is written by a single author with very large, one-off commits, or if the code style is inconsistent and lacks comments, the software might have been thrown together quickly or even vibe-coded (i.e., generated via iterative prompting of AI coding assistants with minimal human review) using AI without thorough human oversight. Because these surface signals also describe many legitimate small projects authored by a single developer, look for stronger positive checks: an automated test suite, continuous integration workflows that exercise those tests on every change, and a pull request history showing merges approved by someone other than the original author. Lack of peer review in code can let subtle errors slip through. Currently, detecting AI-generated code relies largely on these indirect heuristics, but emerging tools such as git-ai [35] aim to track AI authorship at the commit level, annotating which lines were generated by coding assistants. As such tools mature and see broader adoption, they may provide a more transparent and quantitative basis for assessing AI provenance in scientific software. In contrast, when software is developed with rigorous code review practices, it tends to have far fewer bugs [36]. In industry, it’s well documented that having more eyes on code (and more discussion on changes) greatly improves code quality and reliability. So, if you suspect the code has not been reviewed or tested (for example, no issue tracker activity, no tests, and sparse documentation in code), treat the tool with caution. At the very least, you may want to validate its outputs carefully, or favor alternatives that have a stronger record of quality control.

## Conclusions

As researchers increasingly rely on software they did not develop, the ability to critically evaluate tools for sustainability, security, and reproducibility has become essential. While a rich ecosystem of community-developed software exists, not all tools are created equal. Poor choices can compromise results, waste time, or introduce hidden vulnerabilities. By applying the practical tips outlined above, researchers can make informed, confident decisions about the tools they adopt, prioritize robust and well-maintained software, and ultimately contribute to more trustworthy and durable computational biology.
